# Enhanced expression of ROCK in left atrial myocytes of mitral regurgitation: a potential mechanism of myolysis

**DOI:** 10.1186/s12872-015-0038-9

**Published:** 2015-05-09

**Authors:** Huang-Chung Chen, Jen-Ping Chang, Tzu-Hao Chang, Yu-Sheng Lin, Yao-Kuang Huang, Kuo-Li Pan, Chih-Yuan Fang, Chien-Jen Chen, Wan-Chun Ho, Mien-Cheng Chen

**Affiliations:** Division of Cardiology, Department of Internal Medicine, Kaohsiung Chang Gung Memorial Hospital, Chang Gung University College of Medicine, 123 Ta Pei Road, Niao Sung District, Kaohsiung City, 83301 Taiwan; Division of Cardiovascular Surgery, Kaohsiung Chang Gung Memorial Hospital, Chang Gung University College of Medicine, Kaohsiung, Taiwan; Graduate Institute of Biomedical Informatics, Taipei Medical University, Taipei, Taiwan; Division of Cardiology, Chang Gung Memorial Hospital, Chiayi, Taiwan; Department of Thoracic and Cardiovascular Surgery, Chang Gung Memorial Hospital, Chiayi, Taiwan

**Keywords:** Mitral regurgitation, Myocyte, Myolysis, Rho-associated kinase

## Abstract

**Background:**

Severe mitral regurgitation (MR) may cause myolysis in the left atrial myocytes. Myolysis may contribute to atrial enlargement. However, the relationship between Rho-associated kinase (ROCK) and myolysis in the left atrial myocytes of MR patients remain unclear.

**Methods:**

This study comprised 22 patients with severe MR [12 with atrial fibrillation (AF) and ten in sinus rhythm]. Left atrial appendage tissues were obtained during surgery. Normal left atrial tissues were purchased. Immunofluorescence histochemical and immunoblotting studies were performed.

**Results:**

The expression of ROCK2 in the myolytic left atrial myocytes of MR AF patients (*p* = 0.009) and MR sinus patients (*p* = 0.011) were significantly higher than that of the normal subjects. Similarly, the expression of ROCK1 in the myolytic left atrial myocytes of MR AF patients was significantly higher than that of the normal subjects (*p* = 0.010), and the expression of ROCK1 in the myolytic left atrial myocytes of MR sinus patients was higher than that of the normal subjects (*p* = 0.091). Immunofluorescence study revealed significant co-localization and juxtaposition of ROCK2 and cleaved caspase-3 in the left atrial myocytes both in the MR AF group (Pearson’s coefficient = 0.74 ± 0.03) and the MR sinus group (Pearson’s coefficient = 0.73 ± 0.02). Similarly, immunofluorescence study revealed significant co-localization and juxtaposition of ROCK1 and cleaved caspase-3 in the left atrial myocytes both in the MR AF group (Pearson’s coefficient = 0.65 ± 0.03) and the MR sinus group (Pearson’s coefficient = 0.65 ± 0.03). Correlation analysis demonstrated that there was a significant direct relationship between the expression of ROCK2 in the myolytic left atrial myocytes and left atrial diameter in the MR patients (*p* = 0.041; *r* = 0.440). Moreover, the ratio of phosphorylated myosin-binding subunit of myosin light chain phosphatase (pMBS)/total MBS of left atrial tissues was significantly higher in the MR AF group (*p* < 0.04) and the MR sinus group (*p* < 0.04) compared with the normal control group.

**Conclusions:**

The enhanced expression of ROCKs might be involved in the myolysis of the left atrial myocytes of MR patients.

## Background

Valvular heart disease, particularly mitral regurgitation (MR), is one of the major risk factors of heart failure [1] and severe MR portends a poor outcome. Significant left atrial enlargement associated with MR has been known to correlate with a poor prognosis in patients undergoing mitral valve repair or replacement and is often associated with the development of atrial fibrillation (AF) [2]. Atrial fibrillation is the most common cardiac arrhythmia. Previous Western community-based cohort studies revealed that AF increased a two-fold risk in mortality [3, 4]. Furthermore, valvular heart disease with heart failure and AF was linked to increased mortality [5, 6].

Atrial myocardial stretch caused by volume and pressure overload due to significant MR may cause apoptosis. A previous study revealed that apoptosis occurs in the atrial myocytes of patients with mitral valve diseases [7]. Apoptosis plays a critical role in myocyte loss [8]. Indeed, previous studies revealed that myolysis occurs in the atrial myocytes of patients with mitral valve disease [7, 9–11]. Myolysis may contribute to atrial contractile dysfunction and consequently, atrial enlargement as well, which has been reported to be a prognostic factor in patients with MR [2]. However, the underlying mechanisms of myolysis remain unclear.

Growing evidence indicates that Rho-associated kinase (ROCK) activity in human is enhanced in various diseases, including essential hypertension, coronary and cerebral vasospasm, ischemic heart disease, pulmonary hypertension, and heart failure [12–17]. ROCKs may play a role either as a proapoptotic or anti-apoptotic regulator [18, 19]. The ROCKs mediated apoptosis could be either caspase 3-dependent or caspase 3-independent cleavage of ROCKs [19]. However, exactly how ROCK regulates an apoptotic response is not completely understood in many instances, and is likely different depending on the cell type and the apoptotic stimulus [20]. There are two isoforms of ROCKs, ROCK1 and ROCK2 [21, 22]. ROCK2 is distributed mostly in the heart and brain. However, ROCK1 is mainly expressed in the lung, liver, spleen, kidney and testis. Similarly, caspase-3, a key mediator of apoptosis, has a significant role in myocyte apoptosis and is a therapeutic target in heart failure [23–26]. However, the relationship between ROCKs and caspase-3 in the atrial myocytes of heart failure patients due to severe MR remains unknown. Additionally, the relationship between ROCKs and myolysis in the left atrial myocytes of MR patients remain unclear. We hypothesized that there was a positive correlation between myolysis and the expression levels of ROCKs in the left atrial myocytes of heart failure patients due to severe MR. Accordingly, the present study investigated the expression levels of ROCKs and caspase-3 in the left atrial myocytes of heart failure patients due to severe MR.

## Methods

### Patient population

This study examined 22 patients with severe symptomatic MR who had undergone valve operations for heart failure. Exclusion criteria were previous myocardial infarction, febrile disorder, infectious or inflammatory disease, autoimmune disease, malignancy, chronic renal failure (serum creatinine >2.5 mg/dL), acute or chronic viral hepatitis or use of immunosuppressive drugs. Twelve patients had persistent AF [mean (± SD) duration, 47.8 ± 70.2 months; duration range, 1 to 240 months] before surgery (MR AF patients). The sample included ten males and two females with a mean (± SD) age of 67 ± 7 years old (age range, 58 to 81 years old). Ten patients with no history and no records of electrocardiograms of AF before surgery had symptomatic severe MR (MR sinus patients). The sample included two males and eight females with a mean (± SD) age of 56 ± 10 years old (age range, 33 to 68 years old). Informed consent was obtained from all study subjects. The study protocol conforms to the ethical guidelines of the 1975 Declaration of Helsinki and was approved by the Institutional Review Committee for Human Research at our institution. Normal adult left atrial tissue samples were purchased from BioChain Institute, Inc, USA, Novus, USA, and G-bioscience, USA for histochemical and immunochemical studies. These normal atrial tissues were used as the normal controls.

### Echocardiography

Transthoracic echocardiographic examinations were performed on all patients using a 2.5 MHz transducer attached to a commercially available echo Doppler machine (Sonos 7500; Hewlett-Packard; Palo Alto, CA) on the day before valve surgery. Echocardiographic measurements were performed according to the recommendations of the American Society of Echocardiography.

### Hemodynamic measurements

Measurements of left atrial pressure were performed within one month before surgery.

### Specimen storage

Atrial tissue was sampled from the left atrial appendage. After excision, atrial tissues were immediately frozen in liquid nitrogen or embedded in optimal cutting temperature compound, and stored at −80 Celsius to be held for later immunofluorescence staining and immunoblotting.

### Immunofluorescence staining

Frozen tissue sections (5 μm) were fixed for 10 min with 4 % paraformaldehyde and then exposed for 50 min in 5 % BSA (bovine serum albumin), followed by incubation with the corresponding antibodies in the double staining procedures. Primary antibodies included antibodies against ROCK1, ROCK2 (1:50 dilution; Santa Cruz, CA, USA), phalloidin-FITC F-actin (counterstaining for myocyte identification; 1:1000 dilution; Sigma, MO, USA), and cleaved caspase-3 (1:200 dilution; Cell signaling, MA, USA) at 4 °C overnight. For confocal microscope, the secondary detection systems were Alex Fluor 488 (green)/594 (red) goat-anti-mouse IgG (AnaSpec, CA, USA) conjugated with ROCK2, and Alex Fluor 594 (red) goat-anti-rabbit IgG (AnaSpec, CA, USA) conjugated with F-actin and caspase-3 that were diluted 1:500 for 30 min at 37 °C. Nuclei were stained with Hoechst 33258 (1:1000 dilution; Sigma, MO, USA). All images of each specimen were captured and examined at high magnification (600×) using an Olympus FV10I-Oil confocal microscope (Tokyo, Japan).

For immunostaining quantification, atrial samples were analyzed with at least 50 randomly chosen cells per each sample. Cell area, myolytic area, integrated intensities of each antibody (calculated after correction of background noise), percentage of co-localization, and Pearson’s coefficient in each myocyte were obtained and calculated by Olympus Fluoview software (Tokyo, Japan). The expression levels of ROCK2, ROCK1 and cleaved caspase 3 were presented as integrated intensities. Atrial myocytes were scored by morphometry as mildly myolytic if <10 % of the sarcomere content was absent, and moderately-to severely myolytic if >10 % of the sarcomere was absent. For co-localization analysis, the average intensity of each antibody was used as co-localization threshold.

Three normal adult left atrial tissue samples were purchased from BioChain Institute, Inc, USA (76 female, 70 female, and 24 male).

### Western blotting

Tissues extracts were prepared by PRO-PREP™ protein extraction solution (Intron biotechnology, Gyeonggi-do, Korea). Homogenates were centrifuged at 14000 rpm for 30 min at 4 °C to yield supernatants. The concentrations of sample proteins and 3 normal human left atrial proteins [purchased from Novus, USA (77 male), Biochain, USA (62 female), and G-bioscience, USA (24 male)] were determined by the Bradford method (Bio-Rad) according to the supplier’s instructions. Protein extracts were size-fractionated using SDS-PAGE electrophoresis at 7 °C overnight and electro-transferred onto PVDF membranes for 3 h on ice. Membranes were blocked in Tris-buffered saline, with 0.1 % Tween-20 (TBST) and 5 % BSA at room temperature for 2 h. Primary antibodies included phosphorylation level of myosin-binding subunit of myosin light chain phosphatase (pMBS) (1:1000 dilution; Cyclex, Nagano, Japan), total MBS (tMBS) (1:1000 dilution; Cell signaling, MA, USA), ROCK1 (1:500 dilution; Santa Cruz, CA, USA), ROCK2 (1:500 dilution; Santa Cruz, CA, USA), cleaved caspase-3 (1:1000 dilution; Cell signaling, MA, USA) and GAPDH (1:5000 dilution; Millipore, MA, USA) and were used to react with the blots at 4 °C overnight in 5 % BSA. The blots were washed three times in TBST and incubated at room temperature for 1 h with horseradish peroxidase-labeled secondary antibody at dilutions of 1:5000 in TBST containing 5 % BSA. Following three washings, blots were incubated with Immobilon Western chemiluminescent HRP substrate (Millipore, MA, USA). Densitometry analysis was conducted using Quantity One 1-D Analysis Software (Bio-red, Berkeley, California).

### Statistical analysis

Data were presented as means ± SD or SEM. Categorical variables between the MR AF patients, MR sinus patients, and the normal control subjects were compared using chi-square test or Fisher exact test as appropriate. Moreover, continuous variables in the three groups were compared using a Kruskal-Wallis test. Continuous variables between the two groups of study patients were compared with a Mann–Whitney U test. Co-localization analysis between the expression site of ROCKs and the expression site of cleaved caspase-3 in the left atrial myocytes was performed and presented as Pearson’s correlation coefficient. The correlation between the expression levels of ROCKs in the left atrial myocytes and left atrial dimension was performed with the Spearman’s correlation. Finally, statistical analyses were performed using the statistical software program (SPSS version 19.0; SPSS Inc.; Chicago, Illinois, U.S.A.). All *p* values were two-sided, and the level of statistical significance was set at 0.05.

## Results

### Baseline characteristics of patients studied

In this study, all patients were non-smoker, and none had the history of stroke or thromboembolic events. Warfarin was administered to all MR patients with AF. The MR AF patients were older, and included more male patients than MR sinus patients (Table 1). The MR AF patients had a poorer renal function (1.21 ± 0.43 mg/dl vs. 0.72 ± 0.18 mg/dl, *p* < 0.001), and a lower prevalence of dyslipidemia (8.3 % vs. 50.0 %, *p* = 0.043) than the MR sinus patients. The two groups did not differ significantly in term of white blood cell count, body mass index, prevalence of hypertension or diabetes mellitus, and incidence of aortic or tricuspid valve disease. In this study, there was no difference in the severity of heart failure, and advanced heart failure (New York Heart Association functional class ≥3) was diagnosed in 83.3 % of the MR AF patients, and in 90.0 % of the MR sinus patients.

**Table 1:** Baseline clinical characteristics of the study patients

	MR sinus (*n* = 10)	MR AF (*n* = 12)	*p* value
Age (years)	56 ± 10	67 ± 7	0.007
Male (%)	2 (20.0 %)	10 (83.3 %)	0.008
Creatinine (mg/dl)	0.7 ± 0.2	1.2 ± 0.4	<0.001
White blood cell count (10^3^/uL)	6.5 ± 1.9	5.2 ± 1.3	0.069
Body mass index (kg/m^2^)	24.4 ± 2.1	23.9 ± 3.1	0.628
Hypertension (%)	3 (30.0 %)	6 (50.0 %)	0.415
Diabetes mellitus (%)	2 (20.0 %)	3 (25.0 %)	1.000
Dyslipidemia (%)	5 (50.0 %)	1 (8.3 %)	0.043
Heart failure NYHA classification	2.9 ± 0.3	2.9 ± 0.5	0.961
Functional class II (%)	1 (10.0 %)	2 (16.7 %)	
Functional class III (%)	9 (90.0 %)	9 (75.0 %)	
Functional class IV (%)	0 (0.0 %)	1 (8.3 %)	
Advanced heart failure (%)	9 (90.0 %)	10 (83.3 %)	0.571
Aortic valve disease (%)	0 (0.0 %)	2 (16.7 %)	0.481
Tricuspid valve disease (%)	2 (20 %)	7 (58.3 %)	0.099
Left atrial pressure (mmHg)	17.0 ± 8.4	19.3 ± 7.9	0.424
Left atrial diameter (mm)	41.9 ± 5.7	51.7 ± 8.6	0.007
Left atrial ejection fraction (%)	51.6 ± 17.7	38.2 ± 13.6	0.041
Left ventricular end-diastolic diameter (mm)	54.6 ± 5.3	62.4 ± 8.6	0.029
Left ventricular end-systolic diameter (mm)	34.3 ± 5.9	42.5 ± 7.7	0.019
Left ventricular ejection fraction (%)	66.1 ± 7.9	58.5 ± 10.4	0.086
Beta-blockers (%)	1 (10.0 %)	4 (33.3 %)	0.323
Calcium channel blockers (%)	1 (10.0 %)	4 (33.3 %)	0.323
Angiotensin converting enzyme inhibitors or angiotensin II receptor blockers (%)	10 (100.0 %)	10 (83.3 %)	0.481
Statins (%)	1 (10.0 %)	0 (0.0 %)	0.455

Data are presented as mean ± SD or number (percentage).

*NYHA*, New York Heart Association.

The preoperative left atrial size, and left ventricular end-diastolic and end-systolic sizes were significantly larger in the MR AF group than the MR sinus group (Table 1). The left atrial ejection fraction was significantly lower in the MR AF group than the MR sinus group (*p* = 0.043). There was no difference in left atrial pressure and left ventricular ejection fraction between the two groups. Furthermore, the two groups were balanced in terms of use of drugs such as β-blockers, Ca-channel blockers, angiotensin converting enzyme inhibitors or type I angiotensin II receptor blockers, and statins.

### Myolysis and hypertrophy of cardiomyocytes in mitral regurgitation

**Fig. 1:**
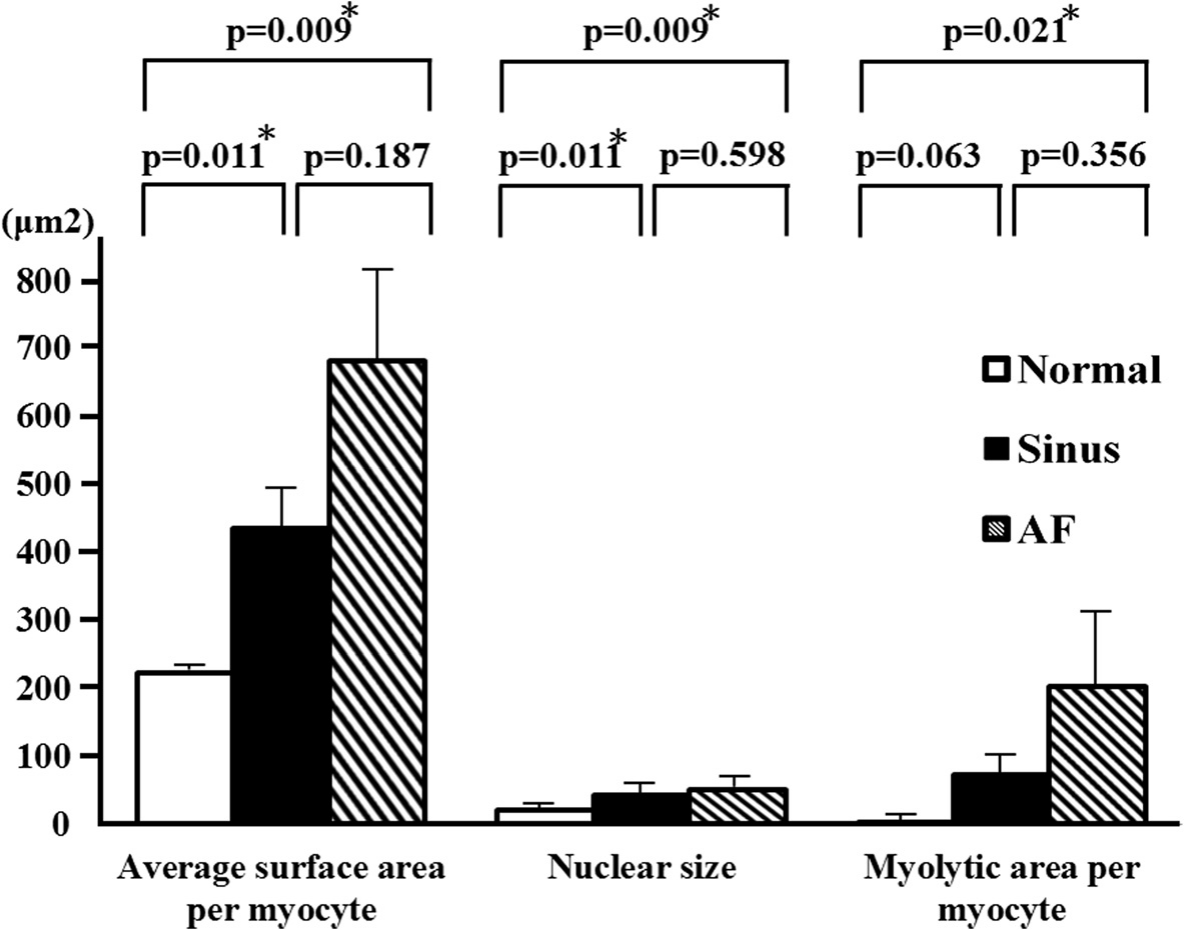
The average cell surface area, average nuclear size, and myolytic area per myocyte in the left atrial tissues of mitral regurgitation patients with sinus rhythm, mitral regurgitation patients with atrial fibrillation (*AF*) and normal control. * *p* < 0.05.

The average cell surface area of myocytes in the left atrial tissue of the MR AF patients (681.6 ± 137.5 vs. 223.1 ± 3.0 μm2, *p* = 0.009) and the MR sinus patients (436.5 ± 65.0 vs. 223.1 ± 3.0 μm2, *p* = 0.011) significantly exceeded the average cell surface area of myocyte in the left atrial tissue of the normal control subjects (Fig. 1). The average cell surface areas of myocytes in the left atrial tissue did not significantly differ between the MR AF group and the MR sinus group (*p* = 0.187). The average nuclear size of myocytes in the left atrial tissues of the MR AF patients (50.4 ± 5.8 vs. 21.4 ± 1.7 μm2, *p* = 0.009) and the MR sinus patients (44.5 ± 4.3 vs. 21.4 ± 1.7 μm2, *p* = 0.011) significantly exceeded the average nuclear size of myocytes in the left atrial tissue of the normal control subjects (Fig. 1). The average nuclear size of myocytes in the left atrial tissue did not significantly differ between the MR AF group and the MR sinus group (*p* = 0.598).

**Fig. 2:**
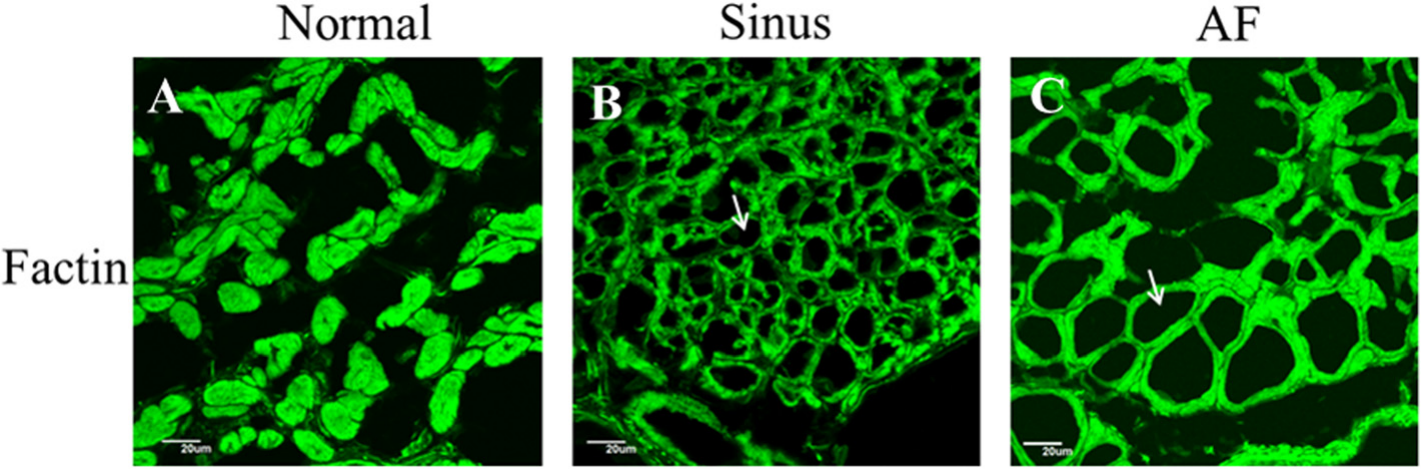
Immunofluorescence images of the normal human adult left atrial tissue sample (*left panel*, **A**), left atrial tissue of mitral regurgitation patients with sinus rhythm (*middle panel*, **B**), and left atrial tissue of mitral regurgitation patients with atrial fibrillation (*AF*) (*right panel*, **C**). Numerous myocytes with perinuclear sarcomere depletion (myolysis) (*arrows*) were found in mitral regurgitation patients with sinus and mitral regurgitation patients with AF. Whereas in normal control atrial sample, myofibrils were organized in a well-aligned and striated network (no myolysis in the myocytes in panel A). Myocyte identification was performed with phalloidin-FITC F-actin (*green color*). Bar = 20 μm.

**Fig. 3:**
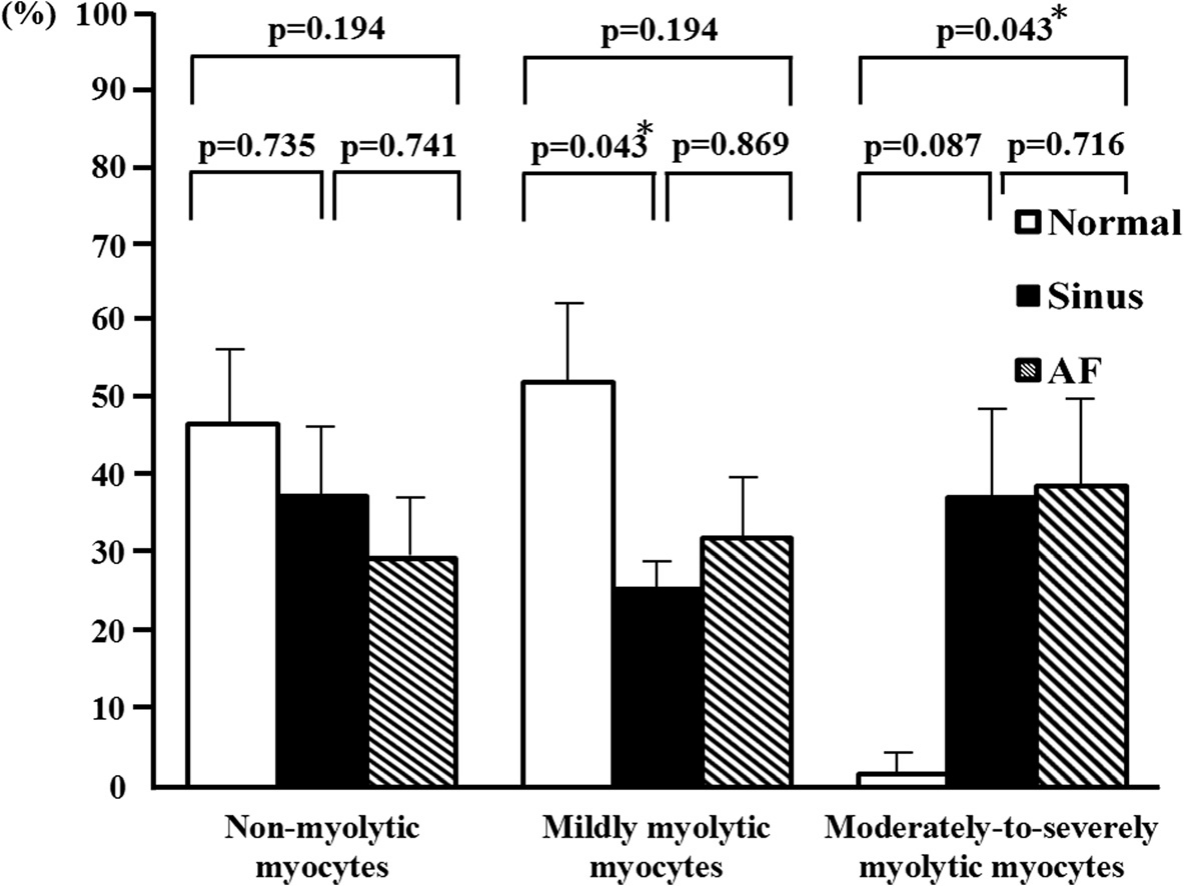
The percentage of atrial myocytes displayed no myolysis, mild myolysis, and moderate-to-severe myolysis in the left atrial tissues of mitral regurgitation patients with sinus rhythm, mitral regurgitation patients with atrial fibrillation (*AF*) and normal control. * *p* < 0.05.

The most prominent change in cellular substructure observed in a significant number of atrial myocytes from patients with MR was the depletion of contractile materials without cell volume loss (myolysis) (Figs. 1 and 2). Loss of contractile materials in many cells was mainly limited to the vicinity of the nucleus, and in some cases, it involved most of the cytoplasm, leaving only a few sarcomeres at the periphery of the cell. The myolytic area per myocyte in the left atrial tissues of the MR AF patients (203.9 ± 89.5 vs. 3.1 ± 1.2 μm2, *p* = 0.021) significantly exceeded the myolytic area per myocyte in the left atrial tissue of the normal control subjects (Fig. 1). The myolytic area per myocyte in the left atrial tissues of the MR sinus patients (73.4 ± 26.8 vs. 3.1 ± 1.2 μm2, *p* = 0.063) exceeded the myolytic area per myocyte in the left atrial tissue of the normal control subjects (Fig. 1). The incidence of atrial myocytes displayed moderate-to-severe myolysis in the left atrial tissues of the MR AF patients significantly exceeded that in the left atrial tissue of the normal control subjects (38.6 ± 11.3 vs. 1.7 ± 1.7 %, *p* = 0.043) (Fig. 3). The incidence of atrial myocytes displayed moderate-to-severe myolysis in the left atrial tissues of the MR sinus patients exceeded that in the left atrial tissue of the normal control subjects (37.1 ± 12.0 vs. 1.7 ± 1.7 %, *p* = 0.087) (Fig. 3).

### Expression of ROCKs and cleaved caspase-3 in atrial myocytes of mitral regurgitation

**Fig. 4:**
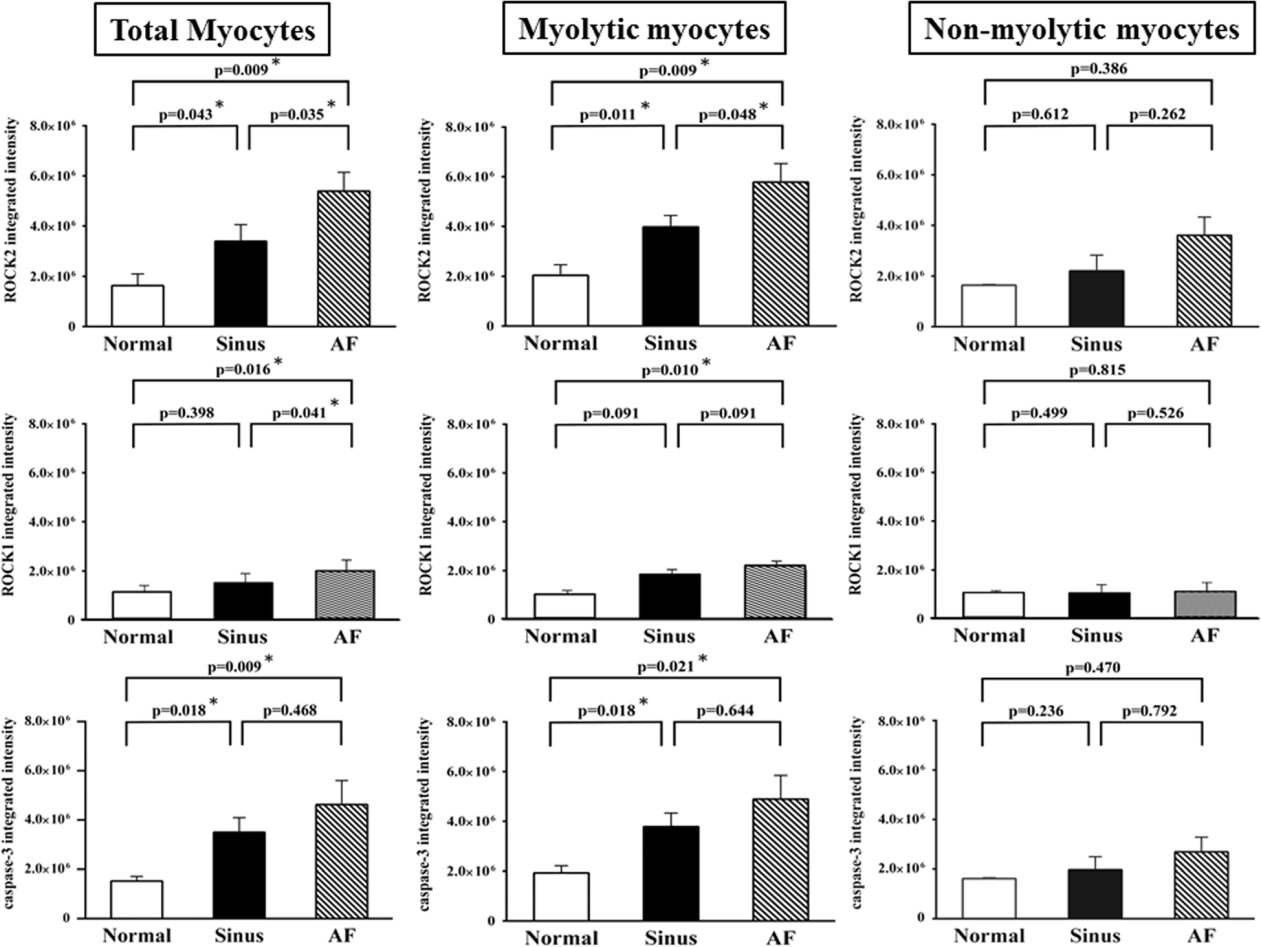
The expression of ROCK2, ROCK1 and cleaved caspase-3 in the myolytic and non-myolytic left atrial myocytes of mitral regurgitation patients with sinus rhythm, mitral regurgitation patients with atrial fibrillation (*AF*) and normal control. * *p* < 0.05.

**Fig. 5:**
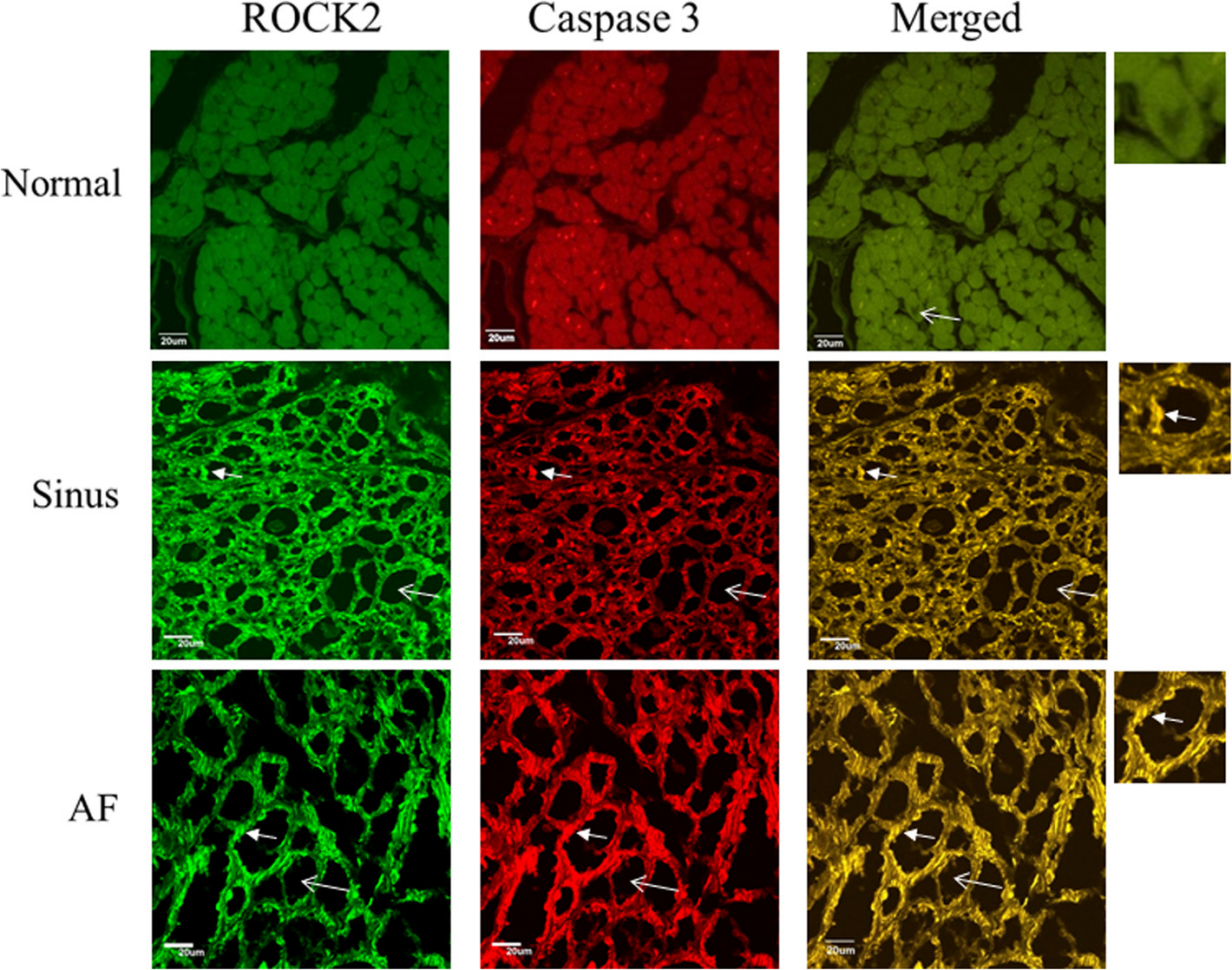
Confocal images of the expression of ROCK2 (*green color*) and cleaved (activated) caspase-3 (*red color*) in the normal human adult left atrial tissue sample (*upper panels*), left atrial tissue of mitral regurgitation patients with sinus rhythm (*middle panels*), and left atrial tissue of mitral regurgitation patients with atrial fibrillation (*AF*) (*lower panels*). Merge pictures revealed co-localization (*insets*, *arrowhead*, *yellow spots*) of expression of ROCK2 and cleaved caspase-3 in the left atrial tissue of mitral regurgitation patients with sinus rhythm (*middle panel*), and mitral regurgitation patients with AF (*lower panel*). Myolysis (*arrow*) was mainly found in mitral regurgitation patients with sinus rhythm and mitral regurgitation patients with AF. Bar = 20 μm.

**Fig. 6:**
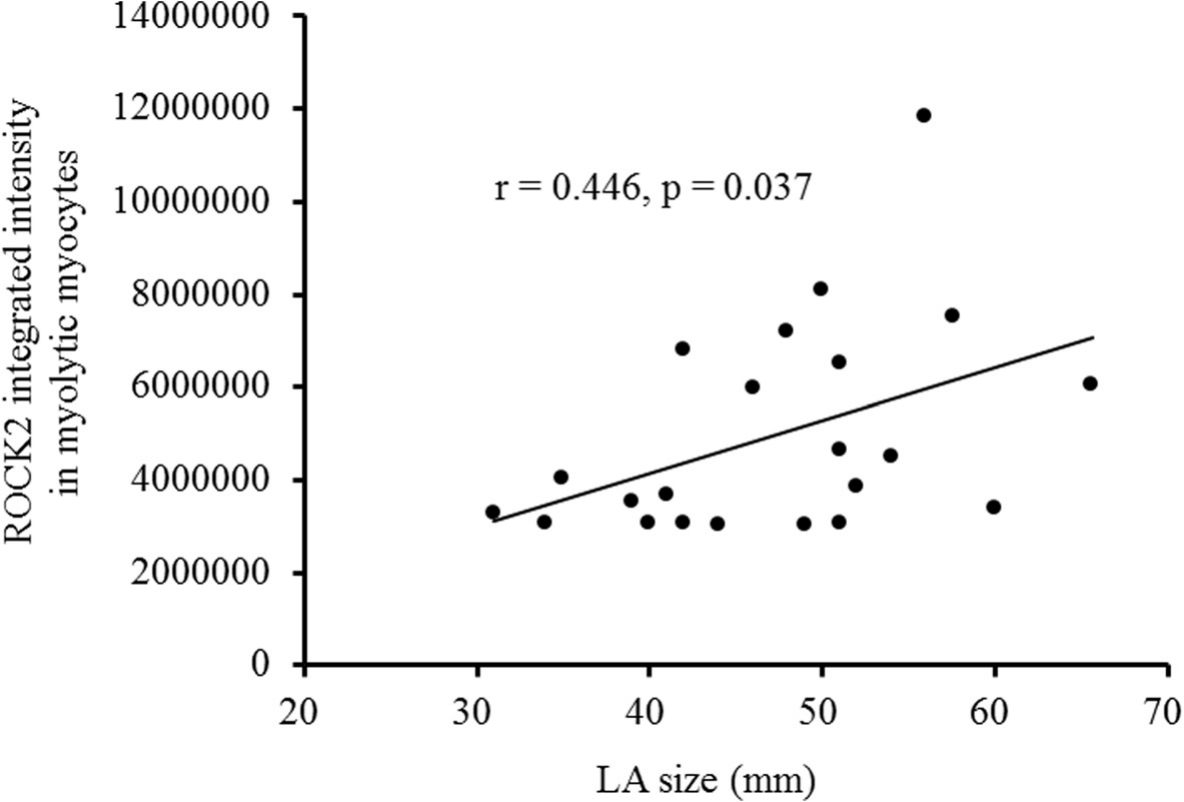
Correlation between the average integrated intensity of ROCK2 in the myolytic left atrial myocytes and the left atrial (*LA*) diameter in the mitral regurgitation patients. Each point represented one patient with mitral regurgitation.

**Fig. 7:**
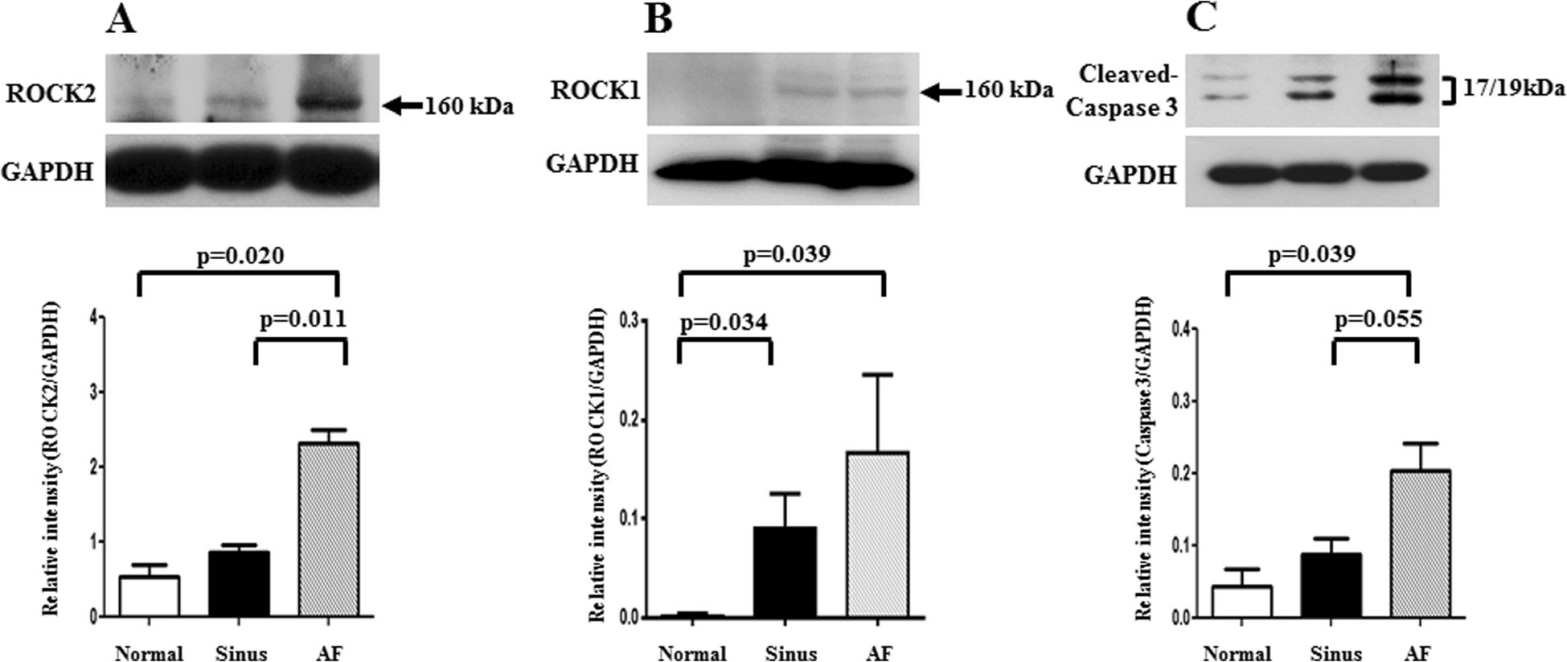
The expression levels of ROCK2 (*left panel*, **A**), ROCK1 (*middle panel*, **B**) and cleaved caspase-3 (*right panel*, **C**) in the left atrial tissue extracts were determined by Western blotting in the normal human adult left atrial tissue samples (*n* = 3), mitral regurgitation patients with sinus rhythm (*n* = 4: small number due to lack of enough tissue samples), and mitral regurgitation patients with atrial fibrillation (*AF*) (*n* = 6).

The expression of ROCK2 in left atrial myocytes of the MR AF patients (5392063.5 ± 754041.5 vs. 1645229.6 ± 33370.6, *p* = 0.009) and the MR sinus patients (3404491.7 ± 651556.7 vs. 1645229.6 ± 33370.6, *p* = 0.043) were significantly higher than the expression of ROCK2 of the normal control subjects (Fig. 4). Of note, the expression of ROCK2 in the myolytic left atrial myocytes of the MR AF patients (5783291.5 ± 745382.4 vs. 1635719.8 ± 465425.9, *p* = 0.009) and the MR sinus patients (3981370.8 ± 457612.4 vs. 1635719.8 ± 465425.9, *p* = 0.011) were significantly higher than the expression of ROCK2 in the myolytic left atrial myocytes of the normal control subjects (Figs. 4 and 5). However, the expression of ROCK2 in the non-myolytic left atrial myocytes of the MR AF patients (3608241.7 ± 720425.7 vs. 2044794.5 ± 422720.6, *p* = 0.386) and the MR sinus patients (2208837.8 ± 624203.5 vs. 2044794.5 ± 422720.6, *p* = 0.612) did not significantly differ from the expression of ROCK2 in the non-myolytic left atrial myocytes of the normal control subjects (Fig. 4). Interestingly, the expression of ROCK2 in the myolytic left atrial myocytes was significantly higher in the MR AF patients than the MR sinus patients (*p* < 0.05) (Fig. 4). Of note, correlation analysis demonstrated that there was a significant direct relationship between the expression of ROCK2 in the myolytic left atrial myocytes and left atrial diameter in the MR patients (*p* = 0.037; *r* = 0.446) (Fig. 6). The expression of ROCK2 protein (normalized to GAPDH) by immunoblotting in left atrial tissues of the MR AF patients (*n* = 6) was significantly higher than the expression of ROCK2 of the normal control subjects (*n* = 3) (2.32 ± 0.18 vs. 0.53 ± 0.16, *p* = 0.020) (Fig. 7a). The expression of ROCK2 protein by immunoblotting in left atrial tissues of the MR sinus patients (*n* = 4) was higher than the expression of ROCK2 of the normal control subjects (*n* = 3) (0.85 ± 0.10 vs. 0.53 ± 0.16, *p* = 0.157) (Fig. 7a). The expression of ROCK2 protein by immunoblotting in left atrial tissues of the MR AF patients was significantly higher than the expression of ROCK2 of the MR sinus patients (*p* = 0.011).

**Fig. 8:**
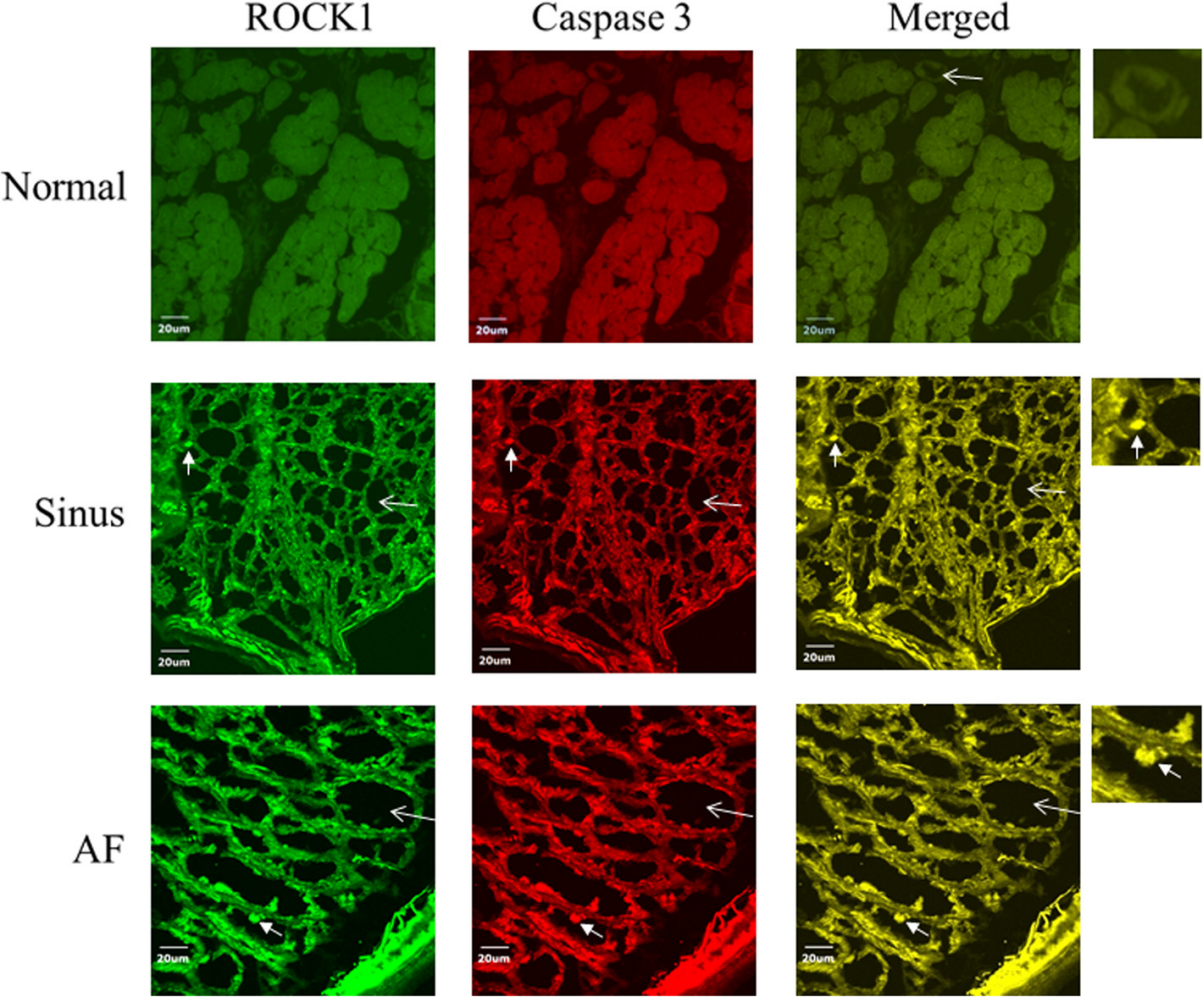
Confocal images of the expression of ROCK1 (*green color*) and cleaved (activated) caspase-3 (*red color*) in the normal human adult left atrial tissue sample (*upper panels*), left atrial tissue of mitral regurgitation patients with sinus rhythm (*middle panels*), and left atrial tissue of mitral regurgitation patients with atrial fibrillation (*AF*) (*lower panels*). Merge pictures revealed co-localization (*insets*, *arrowhead*, yellow spots) of expression of ROCK1 and cleaved caspase-3 in the left atrial tissue of mitral regurgitation patients with sinus rhythm (*middle panel*), and mitral regurgitation patients with AF (*lower panel*). Myolysis (*arrow*) was mainly found in mitral regurgitation patients with sinus rhythm and mitral regurgitation patients with AF. Bar = 20 μm.

The expression of ROCK1 in left atrial myocytes of the MR AF patients was significantly higher than the expression of ROCK1 of the normal control subjects (2033899.5 ± 172865.3 vs. 1132555.5 ± 42027.4, *p* = 0.016) (Figs. 4 and 8). Of note, the expression of ROCK1 in the myolytic left atrial myocytes of the MR AF patients was significantly higher than the expression of ROCK1 in the myolytic left atrial myocytes of the normal control subjects (2173527.6 ± 159745.3 vs. 978383.3 ± 116680.7, *p* = 0.010). However, the expression of ROCK1 in the non-myolytic left atrial myocytes of the MR AF patients did not significantly differ from the expression of ROCK1 in the non-myolytic left atrial myocytes of the normal control subjects (1303403.2 ± 257033.5 vs. 1248082.3 ± 50698.5, *p* = 0.815). Interestingly, the expression of ROCK1 in left atrial myocytes of the MR AF patients was significantly higher than that of the MR sinus patients (2033899.5 ± 172865.3 vs. 1514957.0 ± 231591.3, *p* = 0.041) (Figs. 4 and 8). The expression of ROCK1 in the myolytic left atrial myocytes of the MR AF patients was higher than that of the MR sinus patients (2173527.6 ± 159745.3 vs. 1804745.3 ± 302136.4, *p* = 0.091). The expression of ROCK1 in left atrial myocytes of the MR sinus patients was higher than the expression of ROCK1 of the normal control subjects (1514957.0 ± 231591.3 vs. 1132555.5 ± 42027.4, *p* = 0.398). The expression of ROCK1 in the myolytic left atrial myocytes of the MR sinus patients was higher than the expression of ROCK1 in the myolytic left atrial myocytes of the normal control subjects (1804745.3 ± 302136.4 vs. 978383.3 ± 116680.7, *p* = 0.091) (Figs. 4 and 8). Of note, correlation analysis demonstrated that there was a direct relationship between the expression of ROCK1 in the myolytic left atrial myocytes and left atrial diameter in the MR patients (*p* = 0.057; *r* = 0.422). The expression of ROCK1 protein (normalized to GAPDH) by immunoblotting in left atrial tissues of the MR AF patients (*n* = 6) was significantly higher than the expression of ROCK1 of the normal control subjects (*n* = 3) (0.170 ± 0.078 vs. 0.004 ± 0.003, *p* = 0.039) (Fig. 7b). The expression of ROCK1 protein by immunoblotting in left atrial tissues of the MR sinus patients (*n* = 4) was significantly higher than the expression of ROCK1 of the normal control subjects (*n* = 3) (0.090 ± 0.036 vs. 0.004 ± 0.003, *p* = 0.034) (Fig. 7b). The expression of ROCK1 protein by immunoblotting in left atrial tissues of the MR AF patients was higher than the expression of ROCK1 of the MR sinus patients but the difference did not reach statistical significance (*p* = not significant).

**Fig. 9:**
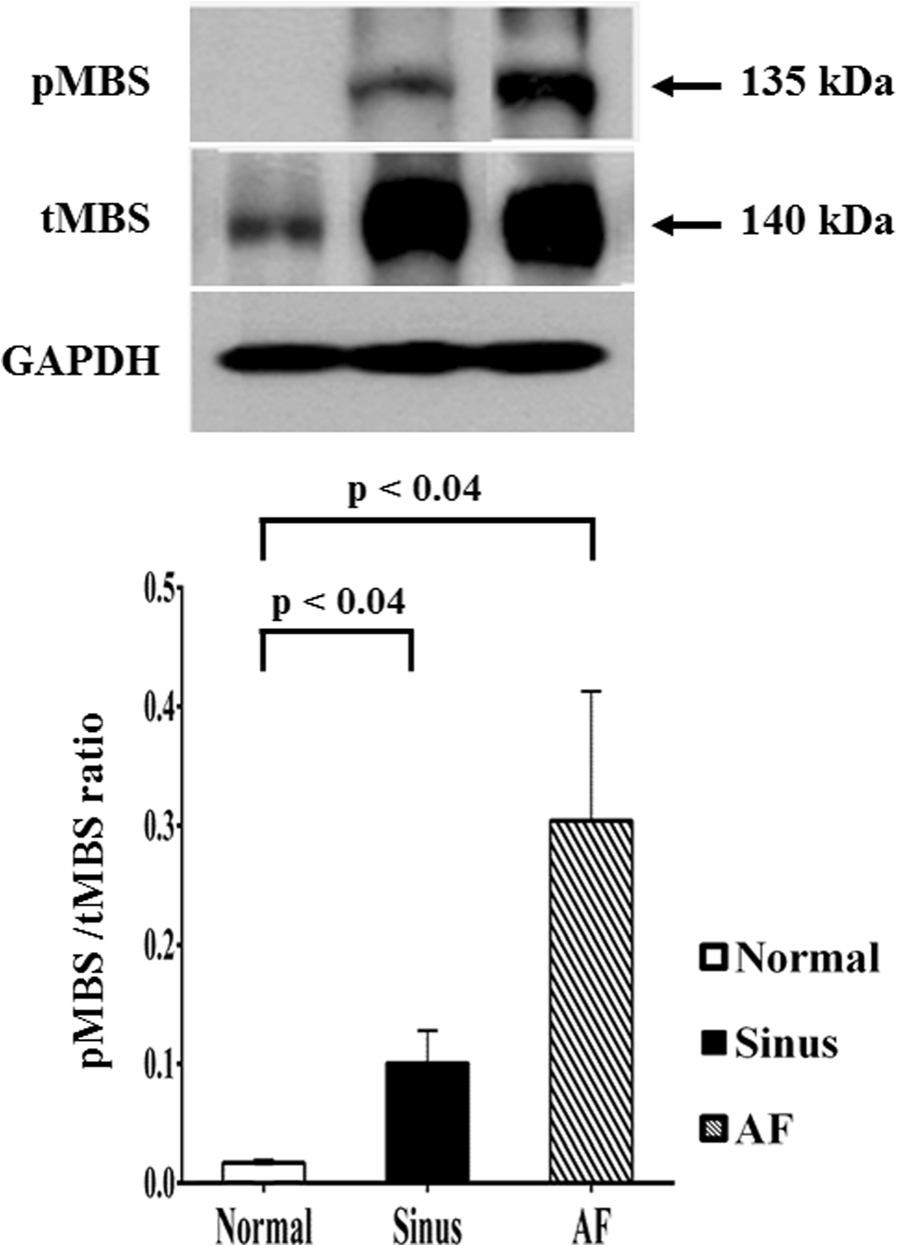
The expression levels of the phosphorylation level of myosin-binding subunit of myosin light chain phosphatase (pMBS), and of the total MBS (tMBS) (normalized to GAPDH) in the left atrial tissue extracts were determined by Western blotting in the normal human adult left atrial tissue samples (*left panel*; *n* = 3), mitral regurgitation patients with sinus rhythm (*middle panel*; *n* = 4: small number due to lack of enough tissue samples), and mitral regurgitation patients with atrial fibrillation (*AF*) (*right panel*; *n* = 6). The ROCK activity is expressed as a relative blot density ratio (pMBS sample density/tMBS sample density).

The ROCK activity is expressed as a relative blot density ratio (pMBS sample density/tMBS sample density) [27]. The ratio of pMBS/tMBS of left atrial tissues was significantly higher in the MR AF group compared with the normal control group (0.30 ± 0.11 vs. 0.02 ± 0.01, *p* < 0.04) (Fig. 9). Similarly, the ratio of pMBS/tMBS of left atrial tissues was significantly higher in the MR sinus group compared with the normal control group (0.10 ± 0.03 vs. 0.02 ± 0.01, *p* < 0.04) (Fig. 9).

The expression of cleaved (activated form) caspase-3 in left atrial myocytes of the MR AF patients (4625762.9 ± 975783.2 vs. 1530603.3 ± 189545.3, *p* = 0.009) and the MR sinus patients (3499251.8 ± 594548.3 vs. 1530603.3 ± 189545.3, *p* = 0.018) were significantly higher than the expression of cleaved caspase-3 of the normal control subjects (Fig. 4). Of note, the expression of cleaved caspase-3 in the myolytic left atrial myocytes of the MR AF patients (4891266.4 ± 952777.4 vs. 1932986.9 ± 280229.3, *p* = 0.021) and the MR sinus patients (3785119.0 ± 540198.5 vs. 1932986.9 ± 280229.3, *p* = 0.018) were significantly higher than the expression of cleaved caspase-3 in the myolytic left atrial myocytes of the normal control subjects (Figs. 4 and 5). However, the expression of cleaved caspase-3 in the non-myolytic left atrial myocytes of the MR AF patients (2691764.1 ± 593821.1 vs. 1621900.3 ± 39281.4, *p* = 0.470) and the MR sinus patients (1970992.5 ± 524326.6 vs. 1621900.3 ± 39281.4, *p* = 0.236) did not significantly differ from the expression of cleaved caspase-3 in the non-myolytic left atrial myocytes of the normal control subjects. The expression of cleaved caspase-3 in the myolytic left atrial myocytes of the MR AF patients did not differ from the expression of cleaved caspase-3 in the myolytic left atrial myocytes of the MR sinus patients (*p* = 0.644). The expression of cleaved caspase-3 protein (normalized to GAPDH) by immunoblotting in left atrial tissues of the MR AF patients (*n* = 6) was significantly higher than the expression of cleaved caspase-3 of the normal control subjects (*n* = 3) (0.20 ± 0.04 vs. 0.04 ± 0.02, *p* = 0.039) (Fig. 7c). The expression of cleaved caspase-3 protein by immunoblotting in left atrial tissues of the MR sinus patients (*n* = 4) was higher than the expression of cleaved caspase-3 of the normal control subjects (*n* = 3) (0.09 ± 0.02 vs. 0.04 ± 0.02, *p* = 0.157). The expression of cleaved caspase-3 protein by immunoblotting in left atrial tissues of the MR AF patients was higher than the expression of cleaved caspase-3 of the MR sinus patients (*p* = 0.055).

Immunofluorescence study revealed a significant co-localization and juxtaposition of the expression of ROCK2 and the expression of cleaved caspase-3 in the left atrial myocytes both in the MR AF group (Pearson’s coefficient = 0.74 ± 0.03) and the MR sinus group (Pearson’s coefficient = 0.73 ± 0.02) (Fig. 5), indicating the existence of potential interaction between ROCK2 and cleaved caspase-3 and confirmation of caspase-dependent activation of ROCK2 in the left atrial myocytes of MR patients, and this interaction might be involved in the pathogenesis of left atrial myolysis in MR patients. Similarly, immunofluorescence study revealed a significant co-localization and juxtaposition of the expression of ROCK1 and the expression of cleaved caspase-3 in the left atrial myocytes both in the MR AF group (Pearson’s coefficient = 0.65 ± 0.03) and the MR sinus group (Pearson’s coefficient = 0.65 ± 0.03) (Fig. 8).

## Discussion

This study of symptomatic severe MR indicated that the expression levels of activated caspase 3 and ROCK2, and ROCK activity were significantly enhanced in the left atrial myocytes of MR patients. Moreover, the enhanced expression of activated caspase 3 and ROCK2 was significantly associated with the presence of myolysis. To the best of our knowledge, this is the first study to demonstrate evidence of the activation of ROCK2 in the myolytic left atrial myocytes of patients with MR.

The most prominent histologic findings from surgical atrial specimens of patients with mitral valve disease are myocyte hypertrophy and myolysis, even in the absence of AF [7, 11, 28, 29]. Myolysis is a degenerative changes accompanied with loss of myofibrils, presence of glycogen granules, accumulation of sarcoplasmic reticulum-like material, and aggregates of mitochondria. The underlying mechanisms of myolysis remain unclear. This study showed that myolysis occurred in 70.6 ± 8.1 % of myocytes in the MR AF group, and in 62.7 ± 10.7 % of myocytes in the MR sinus group.

ROCKs play an important role in many cellular functions, including proliferation, migration, adhesion, contraction, gene expression, and apoptosis [18–22]. Additionally, activity of ROCKs has been reported to be related to the development of many cardiovascular diseases [12–17]. Previous studies showed that upregulation of the RhoA/ROCK pathway contributes to a cellular context that switches-on myosin-mediated contraction, and consequently caspase-3 activation, which is an important mechanism for triggering apoptotic induction [18, 26, 30]. Moreover, ROCKs are important in the regulation of cytoskeleton integrity of cells and actomyosin contractility. In this study, the expression levels of activated caspase 3 and ROCK2, and ROCK activity were significantly enhanced and the expression level of ROCK1 was substantially enhanced in the myolytic left atrial myocytes of MR patients (Figs. 4, 5, 8 and 9). Furthermore, there was a significant co-localization and juxtaposition of ROCK2 and ROCK1 with cleaved caspase-3 in the myolytic left atrial myocytes. However, the expression levels of activated caspase 3, ROCK1 and ROCK2 were not enhanced in the non-myolytic left atrial myocytes. Therefore, this study showed that the interaction between activated caspase 3 and ROCK activity might play a role in the development of myolysis. Interestingly, this study showed that the expression of ROCK2 in the myolytic left atrial myocytes was significantly higher in the MR AF patients than the MR sinus patients (*p* < 0.05). Further studies should be conducted to elucidate the mechanisms of this observation.

### Clinical implications

The study suggested that the interaction between activated caspase 3 and ROCK activity was associated with the development of myolysis in the left atrial myocytes of MR patients. Myolysis is an important mechanism of atrial contractile dysfunction and atrial enlargement, which is an important prognostic factor in patients with MR. The results of this study indicated that inhibition of ROCK2 may be potential candidate target to prevent left atrial contractile dysfunction and left atrial enlargement in patients with MR.

### Study limitations

The limitations of this study should be addressed. Firstly, the sample size of this study was relatively small. However, the results were significant. Secondly, the regulation between activated caspase 3 and ROCK activity was not investigated in this study and our results provide avenues of future study.

## Conclusions

The expression levels of activated caspase 3 and ROCK2, and ROCK activity were significantly enhanced in the left atrial myocytes of MR patients. Significant co-localization and juxtaposition of ROCK2 and cleaved caspase-3 was found in the myolytic left atrial myocytes of MR patients. Moreover, the enhanced expression of activated caspase 3 and ROCK2 was significantly associated with the presence of myolysis. The enhanced expression of ROCK2 might be involved in the myolysis of the left atrial myocytes of MR patients.
